# Your neighborhood matters: an ecological social determinant study of the relationship between residential racial segregation and the risk of firearm fatalities

**DOI:** 10.1186/s40621-023-00425-w

**Published:** 2023-03-13

**Authors:** Abdul R. Shour, Ronald Anguzu, Yuhong Zhou, Alice Muehlbauer, Adedayo Joseph, Tinuola Oladebo, David Puthoff, Adedayo A. Onitilo

**Affiliations:** 1 Marshfield Clinic Cancer Care and Research Center, Clinical Research Institute, Marshfield, WI USA; 2 Department of Oncology, Marshfield Clinic Health System, 1000 N Oak Ave, Marshfield, WI 54449 USA; 3 Division of Epidemiology and Social Sciences, Institute for Health and Equity, Medical College of Wisconsin, Milwaukee, WI USA; 4 Logistics, and Guest Relations, Froedtert Hospital, Milwaukee, WI USA; 5 NSIA-LUTH Cancer Center, Lagos University Teaching Hospital, Lagos, Nigeria; 6 Masters of Sustainable Peacebuilding Program, University of Wisconsin Milwaukee, Milwaukee, WI USA; 7 Marshfield Clinic Research Institute, Marshfield Clinic Health System, 1000 N Oak Ave, Marshfield, WI 54449 USA

**Keywords:** Residential segregation, Dissimilarity index, Firearm fatalities, Income inequality, Community resilience

## Abstract

**Background:**

Firearm fatalities are a major public health concern, claiming the lives of 40,000 Americans each year. While firearm fatalities have pervasive effects, it is unclear how social determinants of health (SDOH) such as residential racial segregation, income inequality, and community resilience impact firearm fatalities. This study investigates the relationships between these SDOH and the likelihood of firearm fatalities.

**Methods:**

County-level SDOH data from the Agency for Health Care Research and Quality for 2019 were analyzed, covering 72 Wisconsin counties. The dependent variable was the number of firearm fatalities in each county, used as a continuous variable. The independent variable was residential racial segregation (Dissimilarity Index), defined as the degree to which non-White and White residents were distributed across counties, ranging from 0 (complete integration) to 100 (complete segregation), and higher values indicate greater residential segregation (categorized as low, moderate, and high). Covariates were income inequality ranging from zero (perfect equality) to one (perfect inequality) categorized as low, moderate, and high, community resilience risk factors (low, moderate, and high risks), and rural-urban classifications. Descriptive/summary statistics, unadjusted and adjusted negative binomial regression adjusting for population weight, were performed using STATA/MPv.17.0; *P*-values ≤ 0.05 were considered statistically significant. ArcMap was used for Geographic Information System analysis.

**Results:**

In 2019, there were 802 firearm fatalities. The adjusted model demonstrates that the risk of firearm fatalities was higher in areas with high residential racial segregation compared to low-segregated areas (IRR.:1.26, 95% CI:1.04–1.52) and higher in areas with high-income inequality compared to areas with low-income inequality (IRR.:1.18, 95% CI:1.00–1.40). Compared to areas with low-risk community resilience, the risk of firearm fatalities was higher in areas with moderate (IRR.:0.61, 95% CI:0.48–0.78), and in areas with high risk (IRR.:0.53, 95% CI:0.41–0.68). GIS analysis demonstrated that areas with high racial segregation also have high rates of firearm fatalities.

**Conclusion:**

Areas with high residential racial segregation have a high rate of firearm fatalities. With high income inequality and low community resilience, the likelihood of firearm fatalities increases.

## Background

Firearm fatalities, defined as any purposeful or accidental death that includes firearms as a vector, are a common type of violent crime in the USA (US), and every year, firearms claim the lives of 40,000 people (Butkus et al. 2018). Death from firearms is a significant public health concern in the USA, which is unique among advanced nations in terms of the level of everyday violence, particularly in the number of civilians killed by gunfire (Hamilton and Kposowa 2015). Homicides (33%) and suicides (63%) account for the vast majority of firearm fatalities, and firearm-related homicide and suicide rates are 25.2 and 8.0 times, respectively, higher in the USA than in other high-income countries in the Organization for Economic Co-Operation and Development (Grinshteyn and Hemenway 2016). A recent nationwide cross-sectional study conducted between 2009 and 2017 discovered an annual average of 34,538 emergency department visits for fatal firearm injuries (Kaufman et al. 2021). Firearm-related injuries are costly to the US healthcare system, placing a significant burden on government insurance and self-paying individuals. From 2006 to 2014, the annual cost of initial hospitalizations for firearm-related injuries was $734.6 million, with Medicaid covering one-third and self-pay patients covering one-quarter of the financial burden (Spitzer et al. 2017). As a result, interventions are needed to reduce firearm fatalities.

To design effective interventions, it is critical to understand the systemic factors with which previous research has established significant associations for higher rates of firearm violence, including fatalities. Such links include state-level gun ownership rates, total homicide rates, and lethal suicidal behavior (Monuteaux et al. 2015; Miller et al. 2012). The rich–poor divide and citizen’s trust in institutions are both associated with increased firearm violence, while government welfare spending is associated with decreased rates of firearm violence, including firearm homicide rates (Monuteaux et al. 2015; Miller et al. 2012; Kim 2019; Tadesse et al. 2020). Meanwhile, researchers found another systemic factor, police violence, to be associated with firearm fatalities for non-White people and people from low-income and less resilient neighborhoods (Zare et al. 2022). Residential racial segregation, which limits opportunities, resources, and the well-being of underserved groups, also contributes heavily to such incidents (Lukachko et al. 2014). Studies have shown that 31% of adults in the US report at least one major discriminatory event in their lifetime and 63% report discrimination daily; furthermore, discrimination has been associated with adverse health outcomes for racial/ethnic minorities (trust in physicians, medication adherence, and receipt of health care) (Cuffee et al. 2013; Shavers et al. 2012; Luo et al. 2012). In addition, people from low-income backgrounds are more likely to experience firearm fatalities than people from high-income neighborhoods (Kang 2016).

While the topic of firearm fatalities has benefitted from increased attention in recent years, some gaps persist in understanding its relationship with certain social determinants of health (SDOH) such as residential racial segregation, income inequality, and community resilience. For example, while Knopov et al. examined the disparity between firearm fatalities between Black and White populations during the period 1995–2015 using the Black–White dissimilarity index, more nuanced understanding about the broader non-White population and SDOH are possible with a resilience framework (Knopov et al. 2019). Wong et al. (2020) examined firearm fatalities at the city level using a hierarchical, and random effects model for 275 urban areas in the USA to find differences could be predicted using racial residential segregation data, but did not account for income inequality, non-Black minority populations, or SDOH. While these studies help to broaden our understanding of racial disparities in firearm homicide in order to inform programs and policies that specifically address the negative consequences of racial segregation, they did not focus on the associations between SDOH (residential racial segregation, income inequality, and community resilience) and the likelihood of firearm fatalities. Despite the impact of firearm fatalities, it is unclear how SDOH such as residential racial segregation, income inequality and community resilience affect firearm fatalities. An analysis that takes into consideration such structural factors and socioeconomic determinants of health can reveal more nuances to guide policy.

Therefore, we investigated the relationship between residential racial segregation and the likelihood of firearm fatalities in Wisconsin, while controlling for potential confounders such as income inequality, and community resilience. We controlled for these confounders because the USA has the highest rate of community gun violence of any established democracy (Wang et al. 2020), and there is an urgent need to find affordable, scalable, and community-led interventions to reduce firearm fatalities and the health consequences that accompany them. We controlled for these covariates as a framework to guide interventions to the man-made tragedy of gun violence, including firearm fatalities, by several sectors and the broader community. These SDOH factors (income inequality and community resilience) will identify existing community assets that will serve as the foundation for future community-led interventions, as well as persons who have avoided firearm fatalities. In the end, our findings will contribute to the body of evidence regarding residential segregation’s effect on firearm fatality disparities, adding much needed nuance in the form of additional social determinants of health.

## Methods

### Data sources

This county-level ecological study analyzed data from the SDOH Database from the Agency for Healthcare Research and Quality (AHRQ) (Agency for Healthcare Research and Quality 2022). The AHRQ’s SDOH database is a project supported by the Patient-Centered Outcomes Research (PCOR) Trust Fund and generates linkable SDOH-focused data for use in PCOR research, informs ways of addressing emergent health challenges, and ultimately contributes to improved health outcomes (Agency for Healthcare Research and Quality 2022). The database was created to make it easier to identify a variety of well-documented, linkable SDOH variables across domains without having to access several source files, hence facilitating our research and analysis. The SDOH database geographically linked multiple datasets such as American Community Survey (ACS) (Census Bureau 2019), and the County Health Rankings (CHR) (Kingery 2018), (Remington et al. 2015), using the county as a unique identifier. Zip code and census tract data were not available across all the databases used in this study. We began by downloading the SDOH Database from the AHRQ website, by county data 2019, five-year estimates. Regarding the number of firearms fatalities for each county (counts), counts data were not accessible in the AHRQ's SDOH Database, and only the rate (ratios) were available. To address this issue, we obtained the 2019 count from the CHR website and linked the number of firearm fatalities in each Wisconsin county to the filtered SDOH Database. The CDC's data on firearm fatalities considered both age and gender in general but single variables were not included in the AHRQ's SDOH county-level database. This is why we were unable to include individual variables (e.g., age, gender) at the ecological level (county). All data were publicly available on the AHRQ website and did not require review from the Institutional Review Board.

The US Census Bureau sponsors the American Community Survey (Census Bureau 2019), which was used to create the residential racial segregation Dissimilarity Index (DI), index of income inequality, and community resilience. Every year, the ACS collects data to offer communities accurate and timely social, economic, housing, and demographic data, and because data are gathered from a sample of the population rather than the entire population, all ACS variables are estimates. Estimates must reflect a geographic area with a population of at least 7000 people in order to be included in ACS 5-year data. The Census Bureau Disclosure Review Board also establishes additional standards to preserve confidentiality and respondent privacy. Data are collected continuously throughout the year and pooled over a calendar year to provide estimates for that year. As a result, ACS estimates represent data collected over time rather than a single point in time (such as in the decennial census, which provides population counts as of April 1 of the census year). ACS 5-year estimates are available as 5-year aggregate files from 2005–2009 to 2016–2020 (as of April 24, 2022). ACS 1-year estimates are available for the same timeframe, but they have greater margins of error than 5-year estimates and, more crucially, are less available for smaller geographic areas and population groups. For these reasons, the SDOH Database relies on ACS 5-year estimates. The SDOH Database county file provides ACS 5-year estimates for every year from 2009 to 2020. Variables were chosen from the 5-year data file whose range ended in that data year for each data collection. For example, the 2019 SDOH data file used in our analysis included variables from the 2015 to 2019 5-year dataset. The SDOH Database contains data at three geographic levels: county, ZIP Code, and the variables we selected to respond to our research question only existed at the county level in 2019 (2015–2019 5-year file).

The County Health Rankings in the US, a Robert Wood Johnson Foundation (RWJF)-sponsored dataset, was also made available in the SDOH database (Kingery 2018; Remington et al. 2015). Since 2010, RWJF has created the CHR variables found in the SDOH Database in partnership with the University of Wisconsin Population Health Institute to provide data on how people in one county compare to people in other counties on a variety of health outcomes, including mortality, as well as health factors of particular relevance to SDOH (e.g., the physical environment, among other areas). CHR data sources include the National Center for Health Statistics—Mortality Files. Years included in the SDOH Database ranged from 2010 to 2020, with counties serving as the geographic level. A missing figure is reported for counties with less than ten firearm fatalities throughout the study's time period (2019) (Population Health Institute 2021). Data on fatalities were submitted to the CDC vital registration systems run by the jurisdictions legally responsible for registering vital events (i.e., births, deaths, marriages, divorces, and fetal deaths) (Population Health Institute 2021). The CDC suppresses all data when there are fewer than 10 firearm deaths (Population Health Institute 2021), so firearms data were reported missing for 13 counties, namely Buffalo, Crawford, Florence, Forest, Green Lake, Iron, Kewaunee, Lafayette, Menominee, Pepin, Richland, Rusk, and Taylor. The CDC suppressed these data for privacy reasons, resulting in missing figures for counties with less than 10 firearm fatalities during the time period, and as a result, the missing data were not all zero and could include any number of fatalities fewer than ten. Due to missing values on these counties, our analysis included a unique 59 counties, and this figure does not represent a representative sample of firearm fatalities throughout the study period. This study's inclusion and exclusion criteria included number of firearm fatalities in Wisconsin in 2019. Firearm fatalities that occurred outside of Wisconsin and study period were excluded.

### Study measures

The study outcome (dependent variable) was the number of firearm fatalities in each county (2019) and was used as a continuous variable. The International Classification of Disease (ICD)-10 codes W32-W34, X72-X74, X93-X95, Y22-Y24, and Y35.0 were used to define firearm fatalities in the annual population (Population Health Institute 2021; Hirsch et al. 2016) The county of residence for the person who died, rather than the county where the death occurred, was used to count firearm fatalities (Population Health Institute 2021). Deaths were counted in the deceased's county of residence. As a result, even if a firearm death occurs across the state, the death is recorded in the individual's home county. The exposure (independent variable) was residential racial segregation as measured by the Dissimilarity Index (2019), and was defined as the degree to which non-White and White residents were distributed across counties (Census Bureau 2019; Sources 2021; Allen et al. 2015; Austin et al. 2019). The index ranges from 0 (complete integration) to 100 (complete segregation), and higher values indicate greater residential segregation (Census Bureau 2019; Sources 2021; Allen et al. 2015; Austin et al. 2019). The residential racial segregation index considered race, but individual variable on race was not included in the AHRQ's SDOH county-level database used in the study. This is why we were unable to include race alone as an individual variable since the majority of the variables in the DI already captured this race measure. For the purposes of this study, we created a new variable that categorizes DI by three quantiles, with cutpoints 1 (low), moderate (2) and high (3), similar to previous analysis (Shour et al. 2022). Covariates included income inequality, community resilience, and rural-urban classification. Income inequality was measured using the Gini index of income inequality, which measures how much a distribution deviates from a proportionate distribution, presented for household income, ranging from zero (perfect equality) to one (perfect inequality),calculated by measuring the difference between a diagonal line (purely proportionate distribution) and the distribution of actual values. The Gini index includes values in the unit interval. The closer the index is to zero (where the area A is small), the more equal the income distribution. The closer the index is to one (where the area A is large), the more unequal the income distribution (Sitthiyot and Holasut 2020). We created a new variable for this analysis that categorizes income inequality into three quantiles, with cutpoints 1 (low), moderate (2), and high (3). Community resilience was defined as the number of individuals who live with various risk factors: income to poverty ratio, single or zero caregiver household, crowding, communication barrier, households without full-time year-round employment, disability, no health insurance, age 65 + , no vehicle access, and no broadband internet access. Community resilience was classified as having 0 risk factors (low risk), 1–2 risk factors (moderate risk), and 3 or more risk factors (high risk) (US Census Bureau 2019). For the objectives of this study, we developed a new variable that categorizes community resilience into three quantiles, with cutpoints of 1 (low), moderate (2), and high (3). Rural–urban classification was measured using the Urban–Rural Classification Scheme for Counties developed by the CDC National Center for Health Statistics (NCHS) in 2013 (Ingram and Franco 2014), defined as metropolitan counties (large central metropolitan, large fringe metropolitan, medium metropolitan and small metropolitan) and nonmetropolitan counties (micropolitan and noncore). These were recategorized into rural (nonmetropolitan counties) and urban (metropolitan counties).

### Analysis

We first ran descriptive/summary statistics on all study measures. Second, we conducted an unadjusted analysis between all study variables and their association with the number of firearm fatalities in Wisconsin (outcome), using negative binomial regression. Third, an adjusted analysis was performed to determine the independent relationship of residential racial segregation and the risk of number of firearm fatalities, adjusting for covariates, and county population weight, using negative binomial regression (incidence rate ratio). STATA/MPv.17.0 (StataCorp. 2021) was used for unadjusted and adjusted analyses, and *P*-values ≤ 0.05 were considered statistically significant. Using ArcMap, we created maps of Wisconsin visually displaying the exposure (racial residential segregation (by DI) and outcome (number of firearm fatalities) to help the reader visualize the disparities. We tested global Moran's I on the number of firearm fatalities and the number of firearms per 100 thousand people. The values are 0.084 and 0.378, respectively. There is a moderate spatial autocorrelation in the distribution of the number of firearms per 100 thousand people. Moran's I for continuous segregation measure is 0.156. However, these are calculated based on the WI county boundary with holes (due to missing values). We could not run spatial models given the spatiality in contiguous county boundaries.

## Results

Table 1 shows descriptive and summary statistics for Wisconsin's 72 counties in 2019. There were 802 firearm fatalities in 2019, with a mean (SD); median of 49 (105), 26. Residential racial segregation was primarily low (33.3%), when compared to moderate (31.9%) and high (31.9%). Income inequality was mostly low (36.1%), followed by moderate (33.3%) and high (30.6%). Community resilience was primarily moderate risk (38.9%) and high risk (31.9%), when compared to low risk (29.2%). Rural areas (63.9%) outnumbered urban regions (36.1%).

**Table 1:** Descriptive and summary statistics (N = 72 counties)

SDH Measures	N	%
Year: 2019	72	100
Residential racial segregation		
Low	24	33.33
Moderate	23	31.94
High	23	31.94
Missing	2	2.78
Income inequality		
Low	26	36.11
Moderate	24	33.33
High	22	30.56
Community resilience		
0 risk factors (Low risk)	21	29.17
1–2 risk factors (Moderate risk)	28	38.89
3 or more risk factors (High risk)	23	31.94
Rural-urban classification		
Urban	26	36.11
Rural	46	63.89
Number of firearms fatalities, mean (SD), median, min–Max, variance	59	49 (105), 26, 10–802, 11068
Total Census County Population weighted, mean (SD), Median, min-max	72	80,427 (135,570), 40,648, 4314–951,226

Table 2 illustrates an unadjusted analysis of SDOH factors associated with the number of firearm fatalities in 2019. The likelihood of firearm fatalities was 1.3 times statistically significantly higher in areas with high residential racial segregation (Coef.: 1.28, 95% CI: 0.72–1.85), and 0.9 times higher in areas with moderate residential racial segregation (Coef.: 0.86, 95% CI: 00.28–1.45), when compared to areas with low residential racial segregation. When compared to areas with low income inequality, the likelihood of firearm fatalities was 1.2 times significantly higher in areas with high-income inequality (Coef.: 1.17, 95% CI: 0.67–1.67). The likelihood of firearm fatalities was 1.9 times significantly higher in areas with high risk community resilience, compared to areas with low risk community resilience (Coef.: 1.86, 95% CI: 1.37–2.36). The likelihood of firearm fatalities was 1.4 times significantly lower in rural areas when compared to urban areas (Coef.: − 1.37, 95% CI: − 1.77 to − 0.97).

**Table 2:** SDOH factors associated with the number of firearm fatalities

SDH measures	Coef	95% CI		Sig
Residential racial segregation				
Low	Ref			
Moderate	0.86	0.28	1.45	***
High	1.28	0.72	1.85	***
Income inequality				
Low	Ref			
Moderate	− 0.25	− 0.74	0.24	
High	1.17	0.67	1.67	***
Community resilience				
0 risk factors (Low risk)	Ref			
1–2 risk factors (Moderate risk)	0.31	− 0.19	0.80	
3 or more risk factors (High risk)	1.86	1.37	2.36	***
Rural-urban classification				
Urban	Ref			
Rural	− 1.37	− 1.77	− 0.97	***

^*^p < 0.05, **p < 0.01, ***p < 0.001

After controlling for income inequality, community resilience, and rural-urban classifications, adjusted model results (Table 3) demonstrate that the risk of firearm fatalities was 1.3 times statistically significantly higher in areas with high residential racial segregation than in areas with low residential racial segregation (IRR.: 1.26, 95% CI: 1.04–1.52). Other SDOH factors in the adjusted model were also significantly associated with an increased risk of firearm fatalities. The risk of firearm fatalities was 1.2 times significantly higher in areas with high income inequality, compared to areas with low income inequality (IRR.: 1.18, 95% CI: 1.00–1.40). Compared to areas with low risk community resilience, the risk of firearm fatalities were 0.6 times significantly higher in areas with moderate risk (IRR.: 0.61, 95% CI: 0.48–0.78), and 0.5 times in areas with high risk (IRR.: 0.53, 95% CI: 0.41–0.68).

**Table 3:** Residential racial segregation and risk of firearm fatalities

SDH measures	IRR	95% CI		Sig
Residential racial segregation				
Low	Ref			
Moderate	1.09	0.89	1.33	
High	1.26	1.04	1.52	**
Income inequality				
Low	Ref			
Moderate	1.12	0.93	1.34	
High	1.18	1.00	1.40	*
Community resilience				
0 risk factors (Low risk)	Ref			
1–2 risk factors (Moderate risk)	0.61	0.48	0.78	***
3 or more risk factors (High risk)	0.53	0.41	0.68	***
Rural-urban classification				
Urban	Ref			
Rural	1.13	0.95	1.34	
Total census County population weighted	1 (Exposure)			

^*^p < 0.05, **p < 0.01, ***p < 0.001

**Fig. 1:**
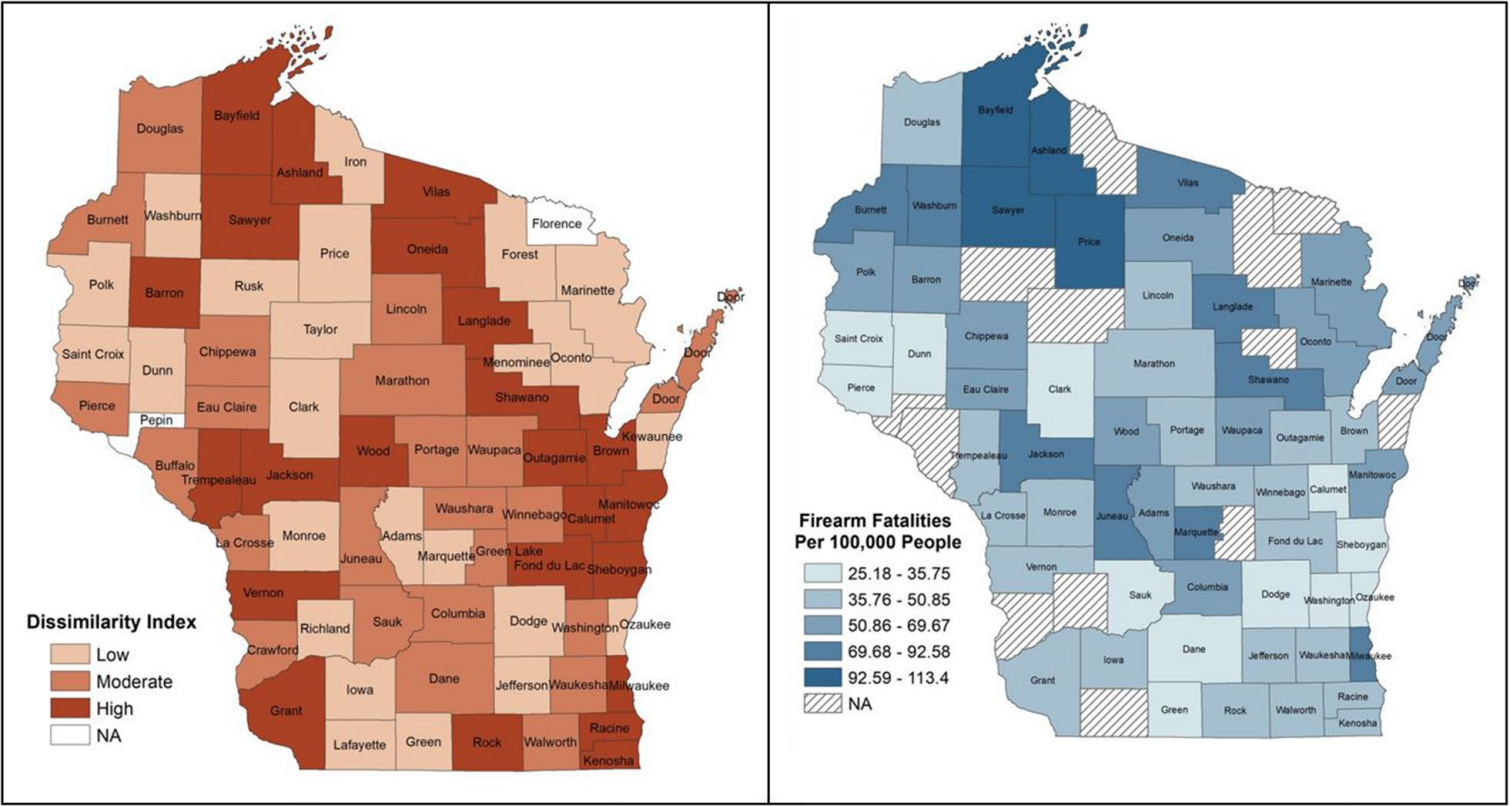
Geographic distribution of residential racial segregation and firearm fatalities in Wisconsin

Figure 1 illustrates a map of Wisconsin that visually depicts the exposure (residential racial segregation) and outcome (firearm fatalities) to help visualize the disparities. Areas with high residential racial segregation also have high rates of firearm fatalities.

## Discussion

This study investigated how SDOH such as residential racial segregation, income inequality and community resilience affect firearm fatalities. We discovered that residential racial segregation and income inequality in Wisconsin were mostly low, compared to moderate and high. However, community resilience was predominantly moderate risk and high risk when compared to low risk, and rural areas outnumbered urban areas. Adjusted model results show that the risk of firearm fatalities is 1.3 times higher in places with high residential racial segregation than in areas with low residential racial segregation after controlling for income inequality, community resilience, and rural-urban classifications. In the adjusted model, other SDOH variables were also significantly associated with an elevated risk of firearm fatalities. When compared to places with low-income inequality, the risk of firearm fatalities was 1.2 times higher in areas with high-income inequality. When compared to low-risk community resilience areas, the risk of firearm fatalities was 0.6 times greater in moderate risk areas and 0.5 times higher in high risk areas. As shown in the map of Wisconsin, areas with high residential racial segregation also have high rates of firearm fatalities, indicating that residential racial segregation influences firearm fatalities. These findings add to the body of knowledge by investigating how structural-level SDOH influence population exposure to firearm fatalities. Our analytical techniques demonstrate that it is possible to combine health agency data with SDOH domains as measured by the DI, income inequality, and community resilience. Using this link to examine the relationship between residential racial segregation and firearm fatalities among county residents, this study discovered that living in a highly racially segregated neighborhood significantly increases the likelihood of firearm fatalities in both unadjusted and adjusted analyses. As shown in this analysis, social determinants of health data can be combined with existing firearm fatalities data to catalyze the investigation of structural, social, and environmental mechanisms such as racial segregation, income inequality, and community resilience—that is, structural mechanisms that may be at play in gun violence victimization. Given these findings, one's residence is important, and data on firearm fatalities should not be viewed in isolation from community contexts.

Our study findings that living in a highly racially segregated area is significantly linked to an increased risk of firearm fatalities are similar to the findings in previous studies that discovered that low-income neighborhoods are more likely to experience violent crime in general than high-income neighborhoods (Kang 2016; Williams and Collins 2001). Another cross-sectional analysis of 51 metropolitan areas in the US from 2013 to 2017 found that structural racism was associated with firearm homicide (Houghton et al. 2021). These studies Tadesse et al. (2020), Zare et al. (2022), Hirsch et al. (2016) contributed significantly to our understanding of violent crime’s social context, including homicide, but did not examine how racial segregation influences firearm fatality while controlling for the novel SDOH measures we used. Our set of determinants, including racial residential segregation, income inequality, and community resilience, constitute macro-level conditions that affect the likelihood of firearm fatalities. Thus, our timely findings demonstrate that one’s county of residence can pose social and physical risks and help address gun violence as a public health issue where prevention and harm reduction can benefit individual and community health.

Our finding that the risk of firearm fatalities was 1.2 times higher in areas with high-income inequality compared to areas with low-income inequality is consistent with the findings of a previous cohort analysis that looked at the association between income inequality and homicide (Rowhani-Rahbar et al. 2019). After controlling for contextual variables of firearm homicide, this analysis demonstrated an association between income inequality and firearm homicide rates across all races/ethnicities, with the association persisting among African-Americans (Rowhani-Rahbar et al. 2019). The study informs policies that target the wealth disparity in order to lower firearm homicide rates and improve public health practices for firearm violence prevention. It did, however, concentrate on the association between county-level income inequality and race/ethnicity-specific firearm homicide rates among people aged 14 to 39. It did not study how SDOH characteristics such as non-White and White residential racial segregation and community resilience affect firearm fatalities across all age groups, nor did it investigate firearm fatalities in general, as we did.

One of our most novel discoveries was that when compared to low-risk community resilience areas, the risk of firearm fatalities was 0.6 times higher in moderate-risk community resilience areas and 0.5 times higher in high-risk community resilience areas, implying that when community resilience is low, the risk of firearm fatalities increases. We measured community resilience by estimating the number of people who live with various risk factors, which was categorized as having 0 risk factors (low risk), 1–2 risk factors (moderate risk), and 3 or more risk factors (high risk). These risk factors include income-to-poverty ratio, single or zero caregiver households, crowding, communication barrier, households without full-time year-round employment, disability, no health insurance, age 65 or older, no vehicle access, and no broadband internet access. An analysis conducted outside of the USA that drew on data from the 2007 and 2009 Citizenship Surveys collected in England (n = 17,572) investigated the role of community resilience such as social capital (bonding, bridging), and discovered that social capital was significantly associated with neighborhood deprivation and self-reported health (Poortinga 2012). After controlling for neighborhood disadvantage, bonding and bridging social cohesion, civic participation, varied socioeconomic relationships, and political efficacy and trust were all associated with community health. While this study defined community resilience at the individual level and recorded various aspects of the social environment, which helps to explain the risk factors for community resilience in our study, ours focused on the ecological level.

### Study's strengths and limitations

Our study has strengths and limitations to consider when interpreting the results. One of the study's significant strengths was its inclusion of new structural measures of SDOH (non-White and White dissimilarity index, income inequality, and community resilience), and the utilization of reputable data sources from the AHRQ which improved the reliability of our findings. The need to understand firearm fatalities necessitates innovative research methods, improved measures, and new approaches for identifying all types of social inequality. However, the first limitation is that firearm fatality was based on the deceased's county of residence rather than the county where the incident occurred, implying that the firearm fatality measure was used as a proxy for exposure to firearm mortality and does not accurately reflect the location of the firearm incident. However, this means that regardless of where a person is, where they live is still a significant determinant. Given the rhetoric surrounding the gun debate in this country, that is an unexpected outcome. It tells us that regardless of where the person encounters violence, their point of origin is important. This contradicts those who believe that certain types of behavior cause violence, and it suggests—at the very least—that how segregated your day-to-day experience in your neighborhood influences your experience of violence whether you leave it or not. Second, our findings do not compare populations who were not reported as victims of firearms mortality, and our analysis excluded the following counties: Buffalo, Crawford, Florence, Forest, Green Lake, Iron, Kewaunee, Lafayette, Menominee, Pepin, Richland, Rusk, and Taylor due to missing firearm fatality data. This missing data implies that our findings cannot be applied to all Wisconsin firearms victims and counties. Furthermore, because our sample was limited to Wisconsin, one of the most racially segregated states in the country (Shour et al. 2021), it cannot be generalized across the entire United States, and more research is needed to examine the relationships between SDOH and firearm fatalities in other states to see if the positive association persists. Third, while we recognize that non-fatal injuries (assault-related injuries, self-harm) are significant public health concerns, particularly among racial/ethnic minorities and young Americans (Kaufman et al. 2021; Fowler et al. 2015), this ecological study did not account for non-fatal injuries and was unable to determine whether these firearm deaths were the result of intentional or unintentional injuries due to how the firearm data was collected. This implies that our findings do not apply to nonfatal firearm injuries, which is particularly concerning given that a nationwide cross-sectional study conducted between 2009 and 2017 discovered an annual average of 85,694 emergency department visits for nonfatal firearm injuries compared to 34,538 deaths from firearm injury (Kaufman et al. 2021). Fourth, our findings are limited to racial rather than ethnic residential segregation because the non-White/White residential segregation measure (DI) used in our study represents racial rather than ethnic residential discrimination. More study is needed to determine whether the positive relationship between residential ethnic segregation (including Hispanics, Latinos, American Indians, and Alaska Natives) and firearm mortality also exists. Fifth, the data used in this study were constrained due to the high frequency of missing data across counties, precluding us from undertaking a multilevel analysis to explore and quantify correlations between variables on a larger scale. To see if DI scores are strongly associated with this amount of variation, future research could use a multilevel approach to assess the level of variation in types of firearm injuries, including fatal and non-fatal injuries and severity, across rural-urban, census tracts, city domains, and ethnic groups. Finally, human-environmental settings, which include the dynamic interaction of psychological, behavioral, socioeconomic, and political protective and risk variables during a person's lifetime, may have an impact on firearm fatalities (Hutchison 2010; Stoddard et al. 2011). Our analysis, however, does not take into account all developmental trajectories formed by human-environmental contexts (social connections, trajectories of hopelessness, and serious violence in impoverished urban youth), nor the interaction between human-environmental context and firearm fatalities from the latent stage through accumulation, pathways, important and sensitive phases, and triggers (Hutchison 2010), (Stoddard et al. 2011).

### Implications for policy and practice

Disparities in firearm fatalities are a serious public health concern that necessitates a multi-stakeholder approach as well as the implementation of tougher interventions to address SDOH that put people at risk of death. Firearm fatalities are widely recognized as a serious public health issue; nevertheless, data for understanding the ecological linkages between SDOH (residential racial segregation, income inequality, and community resilience) and firearm fatalities are notably scarce. For example, a cross-sectional study of 51 US metropolitan statistical regions found that firearm homicide disproportionately affects communities of color and is associated with structural racism indicators such as the White–Black segregation index. (Houghton et al. 2021) While this study contributed to informing public health measures aimed at a specific type of firearm fatality (homicide), it did not account for ecological links between SDOH (non-White and White residential racial segregation, income inequality, and community resilience) and firearm fatalities, as was the case for ours. Our study addressed some of these constraints and discovered that living in areas with high residential racial segregation, high-income inequality, and low community resilience was strongly associated with an increase in the risk of firearm fatalities. Nonetheless, the social and structural mechanisms linking residential racial segregation, income inequality, community resilience, and disparities in firearm fatalities remain largely unknown. There is strong evidence that high firearm fatality rates are statistically related to variations in the prevalence of firearms in the home and the strength of state firearm control legislation, and populations that are most resistant to limits on the availability of firearms are most likely to be affected (Hamilton and Kposowa 2015), though our study did not focus on the impact of firearm control legislation and the impact on people who are most resistant to firearm limits. In light of our finding that areas with high residential racial segregation, high-income inequality, and low community resilience increase the risk of firearm fatalities, our research can inform policymakers regarding the needs of vulnerable populations. More crucially, prioritizing investment initiatives to lower extreme wealth disparity, strengthening community resilience, and minimizing residential racial segregation is essential in mitigating firearm mortality.

## Conclusion

There is a high risk of firearm fatalities in areas with high residential racial segregation, and the risk of firearm fatalities increases when income inequality is high and community resilience is low. This study informs healthcare system-based practice about how the larger social context influences firearm violence disparities and demonstrates how environmental factors (where one lives) influence exposure to firearm fatalities. Multi-level approaches are needed to save lives, reflecting how no single cause or factor of firearm violence exists in isolation (Giffords Law Center to Prevent Gun Violence 2021). Effective gun safety laws and the incorporation of community-based principles (context, partnership processes, interventions, and research and outcomes) (Yonas et al. 2013), (Wallerstein and Duran 2006) will ensure policies and procedures are widely shared and understood, and disparities in firearm mortality are reduced. To avoid unintended negative consequences, policies should be developed with input from a diverse set of stakeholders and applied fairly. Working with policymakers, communities, and organizations, particularly in racially segregated communities, on policy implementation, is more critical than ever.

## Data Availability

While we will be interested in sharing the actual data that were used for analysis, the datasets were publicly available in the AHRQ website and in the County Health Rankings and website repositories: https://www.ahrq.gov/sdoh/data-analytics/sdoh-data.html. https://www.countyhealthrankings.org/explore-health-rankings/county-health-rankings-model/health-factors/social-economic-factors/community-safety/firearm-fatalities?keywords=&f%5B0%5D=type%3Astates&f%5B1%5D=type%3Acounties&year=2019&state=55&tab=1.

## Abbreviations

AHRQ: Agency for Healthcare Research and Quality
CHR: County Health Rankings
ACS: American Community Survey
CDC: Centers for Disease Control and Prevention
DI: Dissimilarity index
ICD: International Classification of Disease
SDOH: Social determinants of health
